# Surfactant replacement and open lung concept – Comparison of two treatment strategies in an experimental model of neonatal ARDS

**DOI:** 10.1186/1471-2466-8-10

**Published:** 2008-07-14

**Authors:** Anne Hilgendorff, Ece Aslan, Thomas Schaible, Ludwig Gortner, Thorsten Baehner, Michael Ebsen, Jochen Kreuder, Clemens Ruppert, Andreas Guenther, Irwin Reiss

**Affiliations:** 1 Dept. of Pediatrics and Neonatology, University of Giessen and Marburg, Germany; 2 Dept. of Perinatology Grosshadern, Ludwig-Maximilian-University, Munich, Germany; 3 Dept. of Pediatrics, University Hospital Mannheim, Germany; 4 Dept. of Pediatrics, University Homburg Saar, Germany; 5 Dept. of Pathology, University of Bochum, Germany; 6 Dept. of Internal Medicine, University of Giessen and Marburg, Germany; 7 Dept. of Pediatric Surgery, Erasmus MC-Sophia Children's Hospital, Rotterdam, The Netherlands

## Abstract

**Background:**

Several concepts of treatment in neonatal ARDS have been proposed in the last years. The present study compared the effects of open lung concept positive pressure ventilation (PPV_OLC_) with a conventional ventilation strategy combined with administration of two different surfactant preparations on lung function and surfactant homoeostasis.

**Methods:**

After repeated whole-lung saline lavage, 16 newborn piglets were assigned to either PPV_OLC _(n = 5) or surfactant treatment under conventional PPV using a natural bovine (n = 5) or a monomeric protein B based surfactant (n = 6).

**Results:**

Comprehensive monitoring showed each treatment strategy to improve gas exchange and lung function, although the effect on Pa_O2 _and pulmonary compliance declined over the study period in the surfactant groups. The overall improvement of the ventilation efficiency index (VEI) was significantly greater in the PPV_OLC _group. Phospholipid and protein analyses of the bronchoalveolar lavage fluid showed significant alterations to surfactant homoeostasis in the PPV_OLC _group, whereas IL-10 and SP-C mRNA expression was tendentially increased in the surfactant groups.

**Conclusion:**

The different treatment strategies applied could be shown to improve gas exchange and lung function in neonatal ARDS. To which extent differences in maintenance of lung function and surfactant homeostasis may lead to long-term consequences needs to be studied further.

## Background

In neonatal acute respiratory distress syndrome (ARDS)-like lung disorders different therapeutic approaches have been introduced in the last years. These include mechanical ventilation strategies as well as exogenous surfactant administration. Various animal studies have focussed this topic and different regimes have been introduced into clinical practice although large clinical trials have not been performed yet [1-3]. As shown in experimental studies, the open lung concept (OLC) as an alternative ventilation strategy, improves gas exchange and reduces ventilator-induced lung injury in models of secondary surfactant deficiency [4,5]. These effects were seen while applying the OLC during high-frequency oscillatory ventilation an positive pressure ventilation [4]. Furthermore, histology and biochemical analyses of bronchoalveolar lavage specimen showed reduced signs of lung injury and pulmonary inflammation in OLC ventilated animals [5]. However, respiratory failure in term neonates is often accompanied by secondary surfactant deficiency, contributing to impairment of lung function in these infants. Thus, exogenous surfactant administration in neonatal ARDS-like lung injury is a clinically well established treatment option [1,6,7]. Even in meconium aspiration syndrome, leading to severe inflammatory-induced lung failure, exogenous surfactant administration is part of therapeutic concepts [8]. Experimentally, the restoration of pulmonary function and gas exchange as well as the amelioration of pulmonary inflammatory processes has been shown [9]. Nevertheless, it has not been extensively investigated whether surfactant therapy in neonatal ARDS attains different effects on gas exchange, lung function, surfactant homoeostasis or pulmonary inflammatory processes compared to the OLC without surfactant replacement, although these effects may lead to differences in short and long term pulmonary outcome following neonatal ARDS. Furthermore, there is still no consensus on ventilation strategy in these infants in combination or without surfactant administration until now [10].

Concerning the choice of the surfactant preparation applied, surfactant preparations with altered protein and phospholipid contents compared to natural surfactant products have gained increasing interest in treatment of ARDS-like lung disorders [9,11-16,20] due to their potential resistance towards surfactant inactivation [17]. Thus, two different surfactant preparations have been chosen for surfactant replacement therapy in the present study: A natural bovine surfactant, frequently used in clinical treatment regimes for neonatal ARDS [18,19] and a modified monomeric SP-B based bovine surfactant, that had recently been demonstrated to improve biological activity of the surfactant preparation compared to standard preparations in a neonatal lung lavage model [20].

Aim of the present study was to compare a treatment strategy applying the OLC without surfactant replacement with a treatment regime using exogenous surfactant administration under conventional ventilation for their effects on gas exchange and lung function variables in a piglet model of neonatal ARDS. Furthermore, different variables of the surfactant system have been assessed using molecular and biochemical analyses of surfactant proteins (SP) and phospholipid composition in order to obtain sensitive markers for lung injury processes. Regarding the impact of different treatment strategies on pulmonary inflammatory processes secondary to lavage-induced lung failure, histologic changes and pulmonary interleukin mRNA expression have been analyzed.

## Methods

### Animal preparation

The experiments were performed according to the Guidelines for the Care and Use of Laboratory Animals of the National Institutes of Health and were approved by local authorities of the animal investigation committee.

Anesthesia was performed in 16 newborn piglets, aged 6 ± 5 days and at a weight of 3.0 ± 0.5 kg (mean ± SD each) with ketamine and midazolam after an intramuscular bolus of ketamine as published previously [9]. The piglets were tracheotomized, a cuffed endotracheal tube (3.0 mm inner diameter; Rüsch, Kernen-Rommelshausen, Germany) was introduced into the trachea and connected to the Evita XL (Dräger, Lübeck, Germany). Animals were ventilated using a pressure-controlled mode with a peak inspiratory pressure (PIP) of 9–12 cm H_2_O, a positive end-expiratory pressure (PEEP) of 2 cm H_2_O, and a respiratory rate of 25–30 cycles/min, a rate of inspiration to expiration of 1:2 at 100% oxygen (FiO_2 _1.0). Under these conditions, normocapnia was observed. A neuromuscular block was induced with pancuromium bromide (0.5 mg/kg i.v.), followed by a continuous infusion with fentanyl (20 μg/kg/h), midazolam (0.3 mg/kg/h) and pancuromium bromide (0.3 mg/kg/h) to provide sedation, analgesia and muscular relaxation. The right common carotid artery was cannulated (20 G, Arrow, Erding, Germany) for continuous blood gas and blood pressure monitoring (Paratrend/Trendcare, Philips Medical, Böblingen, Germany). A double-lumen central venous line (4.0 Fr, Arrow) was placed in the right femoral vein for infusion of fluids and medication. A continuous infusion of 5% dextrose was started (100 mL/kg/d) and all animals received one dose of cefotaxime (100 mg/kg). Body temperature was measured rectally and kept between 38° and 39°C.

### Lavage procedure

Respiratory failure was induced by repeated saline lavage (50 mL/kg; 37°C) as described previously [21]. Lavage procedures were repeated at 3-min interval until partial arterial oxygen pressure (Pa_O2_) was below 10.7 kPa at the following ventilator settings: PIP/PEEP 25/5 cm H_2_O; rate 40 breaths/min; I:E ratio 1:2 and FiO_2 _1.0. A mean of 7 ± 3 lavage procedures were performed per animal and a mean recovery rate of 94% of the lavage fuid was achieved.

### Experimental protocol

Within 10 min after the final lavage, animals were randomly allocated (T = 0 h) to one of the three study groups and ventilated for a period of 5 h. FiO_2 _was kept at 1.0 during the whole experimental procedure. Animals that showed no recovery from lung lavage procedures in the different treatment groups, indicated by a Pa_O2 _< 60 kPa, were excluded from further analyses.

#### PPV_OLC _group

In this group (n = 5), animals with an Pa_O2 _below 13.3 kPa one hour after the whole-lung lavage procedure, were applied to pulmonary recruitment, that was attained according to the protocol previously published by van Kaam et al [5]. The ventilatory rate was increased to 80 breaths per minute with an I:E ratio of 1:1. These settings remained unchanged during the experiment. PEEP was increased up to 15 cm H_2_O and PIP was stepwise increased (5 cm H_2_O each step) to open up the lung. Recruitment of previously collapsed alveoli during this procedure decreased intrapulmonary shunt and thus increased oxygenation [4]. Optimal alveolar recruitment was defined as Pa_O2 _levels ≥ 60 kPa [4]. The level of PIP needed to recruit the lung was accordingly defined the opening pressure (PIP_O_).

After this recruitment procedure, PIP and PEEP were simultaneously decreased in equal steps every 2 to 3 min until Pa_O2 _dropped below 60 kPa, indicating increased intrapulmonary shunting. The level of PEEP at this stage of alveolar collapse was called the closing pressure (PEEP_C_). PEEP was then raised to a level of 2 cm H_2_O above PEEP_C _and PIP was momentarily raised to PIP_O _(about 19 s) to fully recruit the lung. Thereafter, the pressure amplitude was set to keep the partial arterial carbon dioxide pressure (Pa_CO2_) within the target range (4–6 kPa). PEEP only was decreased if there were signs of alveolar overdistension such as increasing Pa_CO2_, decreasing Pa_O2 _or decreasing blood pressure.

#### Surfactant group

In this group, animals were ventilated in the pressure-controlled mode, applying a conventional ventilation strategy, i.e. PIP/PEEP 25/5 cm H_2_O; rate 40 breaths/min; I:E ratio 1:2 and FiO_2 _1.0.

Ten minutes after surfactant depletion with whole-lung lavage animals received either natural bovine surfactant (SF-RI1; Alveofact^®^; n = 5) or a modified surfactant with a monomeric protein B (mon SP-B; n = 6) at a dosage of 100 mg/kg each. Details on surfactant preparations are given below. Surfactant preparations were administered as an intratracheal bolus under continuous chest movements and maintenance of PEEP. Again the pressure amplitude was set to keep the Pa_CO2 _within the target range (4–6 kPa) and PEEP was only decreased if there were signs of alveolar overdistension such as increasing Pa_CO2_, decreasing Pa_O2 _or decreasing blood pressure.

Mean arterial blood pressure, heart rate, ventilator settings, and lung function variables were recorded at the end of the instrumentation period, at the end of the lavage procedure and every 30 minutes thereafter. Although blood gas monitoring was available continuously to provide surveillance of ventilation, data were recorded at these same time points.

### Therapeutic surfactant preparations

Alveofact^® ^(SF-RI1) is a chloroform/methanol extract of bovine lungs containing phospholipids, neutral lipids and the hydrophobic surfactant apoproteins SP-B and SP-C as described previously [22]. The monomeric SP- B surfactant was prepared from SF-RI1 by selective reduction of dimeric SP-B by addition of mercaptoethanol (ME; 50 mg per 2 mL vial) at room temperature for 12 hours and subsequent removement of ME by vacuum extraction. Except for SP-B content subsequent analyses of the obtained surfactant preparations showed no significant differences regarding their phospholipid and apo-protein profile. Both surfactant preparations were provided as lyophilized powder and resuspended in sterile saline 0.9% (Braun) to a final concentration of 60 mg/mL.

### Lung function variables

Tidal volumes, resistance and dynamic compliance were measured by the Evita XL (Dräger, Lübeck, Germany) and related to body weight in order to compensate for different lung volumes. Ventilation efficiency index (VEI, [$VEI=\frac{3,800}{\Delta P\times R\times Pa_{CO_{2}}}$]; [23]) and PIP-PEEP difference (ΔP) were further calculated.

### Bronchoalveolar lavage

At the end of the experiment (T = 5 h) piglets were sacrificed by an overdose of phenobarbitone and bronchoalveolar lavage (BAL) was performed with physiological saline (3 × 50 mL/kg). The percentage of lung lavage fluid recovered was calculated and recovery of BAL fluid was comparable in all animals studied. Samples were centrifuged for 10 min at 300 × *g *at 5°C to remove cells and membranous debris and the supernatant was processed for analyses of surfactant protein and phospholipid concentrations.

### Surfactant protein and phospholipid analyses

#### Protein analysis

Total BAL proteins were calculated using a commercial assay (BCA assay, Pierce, Rockford, IL, USA). SP-B and SP-C were analyzed using ELISA techniques as described previously [24,25].

#### Lipid analysis

Lipids were extracted from BALF with chloroform/methanol [26], and the phospholipid content was determined by spectrophotometric measurement of phosphorus according to the method of Rouser et al. [27]. Individual phospholipid classes were separated by high performance thin-layer chromatography and quantified using scanning densitometry as previously described [28]. Total fatty acids were analyzed by gas-liquid chromatography (Chrompack CP 9001, Varian, Darmstadt, Germany) following acid-catalyled transmethylation into fatty acid methyl ester (FAME) as previously described [29]. For characterization of relative content of large surfactant aggregates (LSA), BALF was centrifuged at 48,000 × g (1 h, 4°C), the pellet was resuspended in 0.9% NaCl and assessed for the PL-content. Recovery of PL in the pellet was used to calculate relative LSA content.

### Histologic processing

After the end of the experiment following the lung lavage procedure, the right lung was perfused with 300 mL of a formaldehyde (4.6%)-glutaraldehyde (0.5%)-solution for approximately 10 minutes. Finally, the trachea was clamped at a PEEP of 10 cmH_2_O, right lungs were removed under maintenance of PEEP and submersed in the above mentioned solution for histomorphologic analyses. Tissue slides were obtained from dependent and non-dependent parts of the right lung. Four tissue slides were analysed from both the upper and lower lung lobe and two slides from the middle lung lobe, respectively. The slides were stained with hematoxylin-eosin (slides of 0.5 μm thickness). Lung histology was evaluated by a pathologist (M.E.), blinded to the animal's group assignment, according to a previously described histologic score [30]. Variables scored for histologic evaluation were atelectasis, alveolar and interstitial inflammation, alveolar and interstitial hemorrhage, alveolar and interstitial edema, necrosis and overdistension. The variables were scored using a four-point scale with no injury corresponding to 0 points and 4 points indicating maximum injury.

### Measurements of interleukin and surfactant protein (SP) -B and -C mRNA expression by realtime PCR

Messenger RNA (mRNA) expression of interleukin (IL) -1β, IL-6, IL-8, IL-10 and SP- B and SP-C was measured in tissue of the left lung lobe using the real-time RT-PCR technique (TaqMan™). Samples were obtained from representative parts of the upper and lower lung lobe.

Primers and probes were designed using Primer Express software (Applied Biosystems, Foster City, USA) following a fixed set of recommendations as described previously [9]. Control PCRs showed no signal for genomic DNA, proving mRNA-specifity. Primers were purchased from Roth (Roth, Karlsruhe Germany), probes from Applied Biosystems, respectively. Lung tissue homogenization was performed in liquid nitrogen and total RNA was then extracted using the acid guanidinium thiocyanate-phenol-chloroform method (Roti Quick, Roth). Total RNA isolation, random primed reverse transcription and real-time PCR was performed following a standardized protocol as described previously [9]. Primer and TaqMan probe sequences are depicted in table 1. Relative quantification was performed using the ΔΔCt method, which results in a ratio of target gene expression and the expression of a housekeeping or reference gene.

**Table 1:** Primer and TaqMan™ probes

**Primer and Probe Description**	**Primer sequences for PCR (forward (F) and reverse (R)) Probe sequences for TaqMan™ analysis**
**Hypoxanthin-guanine-phosphoribosyl-transferase**	F: 5'-TGGAAAGAATGTCTTGATTGTTGAAG-3'
	R: 5'-ATCTTTGGATTATGCTGCTTGACC-3'
	Probe: 5'(VIC)-ACACTGGCAAAAVAATGCAAACCTTGCT-(TAMRA)3'
**β-Actin**	F: 5'-TCATCACCATCGGCAACG-3'
	R: 5'-TTCCTGATGTCCACGTCGC-3'
	Probe: 5'(VIC)-CCTTCCTGGGCATGGAGTCCTGC-(TAMRA)3'
**Interleukin 1 beta (IL-1β)**	F: 5'-GGTTTCTGAAGCAGCCATGG-3'
	R: 5'-GATTTGCAGCTGGATGCTCC-3'
	Probe: 5'(FAM)-AAAGAGATGAAGTGCTGCACCCAAAACCTG-(TAMRA)3'
**Interleukin 6 (IL-6)**	F: 5'-GGGTAGGGAAGGCAGTAGCC-3'
	R: 5'-GAATCCCTCTCCACAAGCG-3'
	Probe: 5'(FAM)-CTTCAGTGGAGTCGCCTTCTCCCTAA-(TAMRA)3'
**Interleukin 8 (IL-8)**	F: 5'-TTCTGCAGCTCTCTGTGAGGC-3'
	R: 5'-GGTGGAAAGGTGTGGAAGTC-3'
	Probe: 5'(FAM)-TTCTGGCAAGAGTAAGTGCAGAACTTCGATG-(TAMRA)3'
**Interleukin 10 (IL-10)**	F: 5'-TTGGAGCTTGCTAAAGGCACT-3'
	R: 5'-CGGCGCTGTCATCAATTTCT-3'
	Probe: 5'(FAM)-CACCTCCTCCACGGCCTTGCTCTT-(TAMRA)3'
**Surfactant protein B (SP-B)**	F: 5'-TCC GCT GGT CGT TGA TCA C-3'
	R: 5'-GTT TGC ACA GGC CCA AGT G-3'
	Probe: 5'(FAM)-CAG AGC CAA ATG AAC CTG AAG GCC ATC-(TAMRA)3'
**Surfactant protein C (SP-C)**	F: 5'-CAC CTT CTC CAT TGG CTC TAG TG-3'
	R: 5'-ATA CTC TGC GGA GAC ATC TTC ATG-3'
	Probe: 5'(FAM)-TGA CTA CCA GCG GCT CCT GAT TGC C-(TAMRA)3'

For housekeeping genes β-actin (A) and hypoxanthin-guanine-phosphoribosyl-transferase (HPRT) were chosen and validated by variation analyses in all samples.

As β-actin showed both no differences between different experimental groups and the lowest variation in all samples, it was chosen as reference gene for further analysis.

### Statistical analyses and data presentation

Results are given as mean and standard deviation (SD). Results of real-time PCR analyses are given in arbitrary units and were normalized to housekeeping gene expression. Data analysis was performed using SPSS for Windows Version 6.1.3. As normal distribution has been shown, two-way ANOVA with repeated measurements was performed for analysis of lung function variables over the time course. In order to compare outcome levels between the groups differences from T = 5 - T = 0 were calculated and one-way ANOVA was performed with posthoc Scheffé. Results of the histologic evaluation as well as from determination of lavage specimen and real-time PCR were analyzed using one-way ANOVA with posthoc Scheffé. Calculated p-values are given in the result section.

## Results

All animals survived during the study period. There were no statistical significant intergroup differences in age, weight or number of lavages needed to induce the lung injury. No air leaks were observed within the study period. Blood gas values and lung function variables before and immediately after lavage were comparable in the three treatment groups (Table 2a). One animal of the natural surfactant group and 2 animals of the mon SP-B surfactant group did not recover after surfactant administration and required intensified ventilation (OLC) in order to reach Pa_O2 _levels ≥ 60 kPa. As this alteration in treatment strategy was not comparable with the other study groups, these animals were therefore excluded from further analysis. Thus, 13 animals were available for analyses.

**Table 2:** Gas exchange and lung function variables

**a. Gas exchange and lung function variables at different time points**							
	**Pa_o2_**	**Pa_CO2_**	**C_dyn_/kg**	**VT/kg**	**Res**	**VEI**	**ΔP**

**Healthy (H)**							
PPV_OLC_	74.21 ± 16.75	5.67 ± 2.05	1.94 ± 0.89	16.0 ± 4.8	37.62 ± 8.09	0.29 ± 0.17	10.20 ± 0.45
mon SP-B	64.11 ± 34.10	3.75 ± 0.75	2.56 ± 0.50	20.4 ± 2.7	40.50 ± 8.63	0.35 ± 0.08	10.00 ± 0.00
nat SF	78.44 ± 15.09	5.29 ± 1.95	2.12 ± 0.87	19.2 ± 7.0	37.88 ± 7.43	0.29 ± 0.14	10.25 ± 0.50
**Lavaged (T = 0)**							
PPV_OLC_	6.70 ± 3.04 **	6.95 ± 2.84	0.56 ± 0.23 *	11.3 ± 2.6	52.43 ± 13.53 *	0.10 ± 0.06 *	20.00 ± 0.00
mon SP-B	9.24 ± 0.79*	3.86 ± 1.64	1.21 ± 0.42 *	21.0 ± 5.0	63.28 ± 15.30 *	0.11 ± 0.02 **	20.00 ± 0.00
nat SF	8.16 ± 1.26 **	5.57 ± 1.35	0.88 ± 0.35 *	16.3 ± 5.4	50.25 ± 11.95 *	0.10 ± 0.04 *	20.00 ± 0.00
**1 h**							
PPV_OLC_	9.68 ± 2.26	6.91 ± 2.07	0.57 ± 0.20	11.3 ± 4.5	47.50 ± 12.90	0.09 ± 0.04	20.00 ± 0.00
mon SP-B	38.60 ± 24.14	3.83 ± 2.84	1.52 ± 0.66	22.9 ± 8.4	58.55 ± 14.04	0.16 ± 0.09	19.50 ± 1.00
nat SF	56.86 ± 14.62	3.75 ± 1.13	1.50 ± 0.43	23.1 ± 6.2	53.98 ± 14.52	0.14 ± 0.05	20.00 ± 0.00
**2 h**							
PPV_OLC_	71.45 ± 20.29	4.49 ± 1.97	1.03 ± 0.38	10.2 ± 2.2	34.34 ± 8.13	0.33 ± 0.20	12.60 ± 1.82
mon SP-B	64.87 ± 29.48	3.51 ± 0.91	1.53 ± 0.10	16.1 ± 7.0	47.80 ± 20.69	0.33 ± 0.27	13.75 ± 3.86
nat SF	75.18 ± 19.67	4.12 ± 0.41	1.39 ± 0.31	18.6 ± 4.0	45.20 ± 7.52	0.16 ± 0.02	17.75 ± 1.71
**3 h**							
PPV_OLC_	74.08 ± 12.99	5.19 ± 2.35	1.13 ± 0.75	9.9 ± 3.0	34.76 ± 10.65	0.30 ± 0.19	12.40 ± 2.07
mon SP-B	59.05 ± 33.81	4.77 ± 1.11	1.23 ± 0.14	14.7 ± 4.1	50.63 ± 14.95	0.19 ± 0.12	14.00 ± 3.74
nat SF	72.32 ± 17.01	4.81 ± 0.55	1.15 ± 0.30	17.1 ± 5.0	43.85 ± 9.29	0.15 ± 0.04	17.00 ± 0.82
**4 h**							
PPV_OLC_	70.09 ± 12.32	5.54 ± 1.67	1.11 ± 0.73	9.7 ± 2.4	35.46 ± 7.11	0.26 ± 0.15	12.60 ± 3.21
mon SP-B	60.05 ± 33.26	4.73 ± 1.28	1.21 ± 0.15	14.8 ± 3.4	53.70 ± 19.89	0.18 ± 0.11	14.50 ± 3.87
nat SF	56.53 ± 11.97	5.37 ± 0.53	1.05 ± 0.27	15.5 ± 2.8	44.18 ± 11.66	0.14 ± 0.04	16.50 ± 0.58
**5 h**							
PPV_OLC_	71.61 ± 9.23	5.25 ± 1.01	0.97 ± 0.51	9.6 ± 3.2	35.78 ± 8.46	0.25 ± 0.14	12.80 ± 3.27
mon SP-B	60.38 ± 33.21	5.49 ± 1.17	1.04 ± 0.22	13.1 ± 3.7	51.93 ± 14.55	0.16 ± 0.08	14.0 ± 4.69
nat SF	48.94 ± 20.65	5.20 ± 0.52	1.01 ± 0.25	15.0 ± 2.2	50.55 ± 13.11	0.12 ± 0.04	17.00 ± 0.82

**b. Overall differences in gas exchange and lung function variables (T5 - T0)**							

	**Pa_o2_**	**Pa_CO2_**	**C_dyn_/kg**	**VT/kg**	**Res**	**VEI**	**ΔP**

**T5 - T0**							
PPV_OLC_	64.90 ± 9.22	-1.71 ± 2.57	0.42 ± 0.40	-2.75 ± 0.96	-17.2 ± 5.43	0.19 ± 0.078 */§	-7.2 ± 3.27
mon SP-B	51.14 ± 33.47	1.63 ± 1.27	-0.18 ± 0.42	-8.0 ± 4.24 *	-11.35 ± 13.10	0.043 ± 0.07	-6.0 ± 4.69
nat SF	40.78 ± 20.49	-0.38 ± 1.71	0.13 ± 0.27	-1.25 ± 3.59	0.3 ± 5.91	0.02 ± 0.06	-3 ± 0.82

mean ± SD; open lung concept positive pressure ventilation (PPV_OLC_; n = 5); surfactant treatment under conventional positive pressure ventilation: natural bovine surfactant (Alveofact^®^; nat SF; n = 4), monomeric surfactant protein B based surfactant (mon SP-B; n = 4). * p < 0.05; ** p < 0.01 vs initial values (healthy; H). Further levels of significance (two-way ANOVA) are given in detail in the text.

* p < 0.05 vs nat SF

§ p < 0.05 vs mon SP-B

No animal included for further analyses showed signs of hemodynamic compromise. Data on hemodynamics were not significantly different between the groups.

### Gas exchange and lung function

**Figure 1:**
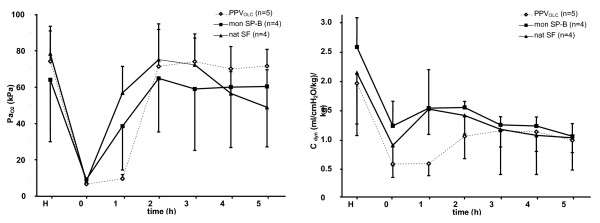
**Gas exchange(Pa_O2_) and lung function (C_dyn_)**. Time course of Pa_O2 _(left panel) and dynamic compliance (C_dyn_; right panel) over the observation period. Induction of lung injury by repetitive lavage procedures (T = 0) is followed by open lung concept positive pressure ventilation (PPVOLC; n = 5) or surfactant treatment under conventional positive pressure ventilation with a modified monomeric surfactant protein B surfactant (mon SP-B; n = 4) or a natural bovine surfactant (Alveofact^®^; nat SF; n = 4). Results are given as mean and standard deviation.

After induction of lung injury, Pa_O2_, dynamic compliance and VEI were significantly reduced in all animals (see figure 1), whereas the resistance increased significantly. Pa_CO2 _remained largely unchanged.

Mean opening pressure was 35 ± 4 cm H_2_O, mean closing pressure 8 ± 3 cm H_2_O in the PPV_OLC _group.

After experimental intervention (OLC, surfactant administration), Pa_O2_, tidal volumes, difference of PIP-PEEP (ΔP), resistance, VEI (p < 0.001 each) and dynamic compliance (p = 0.01) significantly improved over the time course in each treatment group (two-way ANOVA; Table 2a).

Regarding differences between the treatment groups, two-way ANOVA testing revealed a significant difference with respect to Pa_O2 _(p < 0.001; figure 1) and PaCO_2 _(p < 0.05) levels over the time course with declining effects in the nat SF group 3 hours after intervention. Comparing the relative change of the variables over the study period (T = 5 - T = 0), gas exchange variables showed a response to each experimental strategy and the overall change did not differ between the groups (Table 2b).

Comparable results were found for the development of the dynamic compliance and the ΔP over the study period with a significant difference between the study groups: Dynamic compliance could be shown to increase up to two-fold, with declining effects in the surfactant groups after 3 hours (figure 1), whereas ΔP could be decreased in all experimental groups during the observation period with most pronounced effects in the PPV_OLC _and the mon SP-B group (p = 0.001). Nevertheless, changes in dynamic compliance or ΔP over the study period (T = 5 - T = 0) were not statistically significantly different between the groups. Mean airway pressure was found to be significantly higher in the PPV_OLC _group compared to both surfactant treated groups (p = 0.015; data not shown).

The tidal volumes achieved under different ventilation strategies were significantly lower in the PPV_OLC _group over the whole study period compared to both surfactant groups (p = 0.002). Relative changes of tidal volumes (T = 5 - T = 0) showed a significant decline in the mon SP-B group when compared to the nat SF group (p < 0.05 vs nat SF).

**Figure 2:**
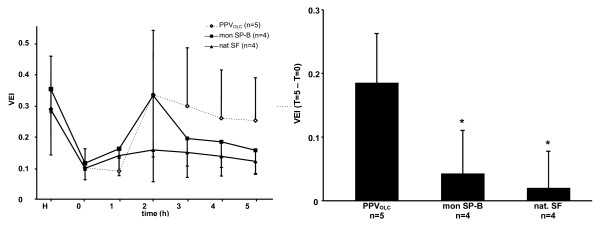
**Ventilation efficiency index (VEI)**. *Left panel*. Time course of ventilation efficiency index (VEI) over the observation period. Induction of lung injury by repetitive lavage procedures (T = 0) is followed by open lung concept positive pressure ventilation (PPV_OLC_; n = 5) or surfactant treatment under conventional positive pressure ventilation with a natural bovine surfactant (Alveofact^®^; nat SF; n = 4) or a modified monomeric surfactant protein B surfactant (mon SP-B; n = 4). *Right panel*. Difference of VEI over the study period (T5-T0). Study groups: open lung concept positive pressure ventilation (PPV_OLC_; n = 5); surfactant treatment under conventional positive pressure ventilation with a modified monomeric surfactant protein B surfactant (mon SP-B; n = 4) or a natural bovine surfactant (Alveofact^®^; nat SF; n = 4). Results are given as mean and standard deviation. * p < 0.05 vs PPV_OLC_.

Improved VEI were found in all treatment groups but differed in its time course between the groups (p = 0.015; figure 2). Most pronounced effects were seen in the PPV_OLC _and the mon SP-B group, although effects decreased in the mon SP-B group towards the end of the observation period (figure 2). The relative change of the VEI over the observation period (T = 5-T = 0) was found to be significantly greater in the PPV_OLC _group compared to the mon SP-B and nat SF treated group (p < 0.05; figure 2).

### Lung histology

**Figure 3:**
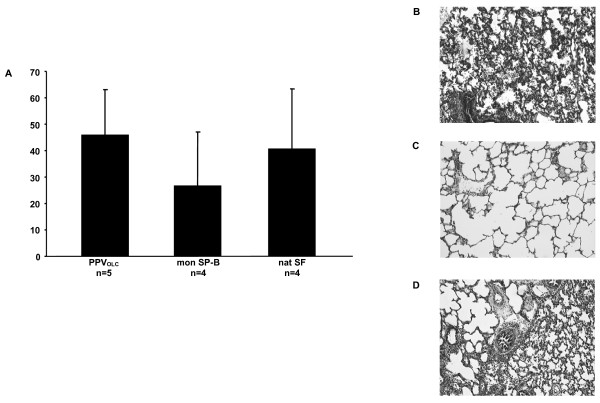
**Histological sum scores (upper, middle and lower right lung lobe)**. Histologic sum scores (atelectasis, alveolar and interstitial inflammation, alveolar and interstitial hemorrhage, alveolar and interstitial edema, necrosis and overdistension) of upper, middle and lower right lung lobe using a four-point scale with no injury corresponding to 0 points and 4 points indicating maximum injury (**A**). Comparison of the groups showed no significant differences. Hematoxylin-eosin staining of exemplary histologic slides showing dystelectasis and neutrophiles in nearly all alveoli in the PPV_OLC _group (**B**), regularly ventilated lung parenchyma in the monomeric SP-B group (**C**) and dystelectasis and neutrophiles in some of the alveoli in the natural SF group (**D**; hematoxylin-eosin, magnification 100×). *Study groups: *open lung concept positive pressure ventilation (PPV_OLC_; n = 5); surfactant treatment under conventional positive pressure ventilation with a modified monomeric surfactant protein B surfactant (mon SP-B; n = 4) or a natural bovine surfactant (Alveofact^®^; nat SF; n = 4). Results are given as mean and standard deviation.

Lung histology was examined in tissue slices of the right upper, middle and lower lung lobes. All study groups revealed significantly higher histologic scores in the upper and lower lung lobe compared to the middle lung lobe (p < 0.05, data not given in detail). Figure 3 shows the histological sum score of the different treatment groups. Although there was a tendency towards lower sum scores in the mon SP-B group (27 ± 20) compared to the PPV_OLC _group (46 ± 17) and the nat SF group (41 ± 23) there was no significant difference.

### Surfactant protein (SP) and phospholipid analyses

Total phospholipid and total protein content, phospholipid class profiles, total lipids fatty acid profiles, recovery of large surfactant aggregates and surfactant proteins (SP)-B and -C, were determined in cell depleted BAL specimen and are displayed in table 3. Total PL concentrations were elevated up to 3-fold in both surfactant groups with significant differences between the nat SF group compared to the PPV_OLC _group (p = 0.012; table 3). Total protein content was not different between the study groups, resulting in an increase of the phospholipid-to-protein ratio ratio in both surfactant groups compared to the PPV_OLC _group (nat SF p = 0.05; mon SP-B p = 0.09). Regarding phospholipid composition, the relative content of phosphatidylcholine and phosphatidylglycerol was found to be significantly increased in both surfactant groups (p < 0.05 vs PPV_OLC_) and reached near normal levels. Accordingly, the relative content of phosphatidylserine, phosphatidylinositol, phosphatidylethanolamine and sphingomyelin decreased in the mon SP-B and nat SF groups with significant effects for phosphatidylserine (p < 0.001 vs PPV_OLC_). Analyses of the total fatty acids revealed a significantly increased relative content of palmitic acid in both surfactant groups (p < 0.01 vs PPV_OLC_; table 3) whereas levels of polyunsaturated fatty acids and all unsaturated fatty acids decreased significantly (p < 0.01 vs PPV_OLC_). Levels of arachidonic acid decreased in both surfactant groups compared to the PPV_OLC _group, eicospentaenoic acid increased in the nat SF treated group (p < 0.01 vs PPV_OLC_).

**Table 3:** Phospholipid and surfactant protein analyses of lavage specimen

**group**	**n (piglets)**	**PL [μg/ml]**	**PPQ**	**LSA [% PL]**	**SP-B/PL [% (w/w)]**	**SP-C/PL [% (w/w)]**	**PC [%]**	**PG [%]**	**PS [%]**	**PI [%]**
**PPV_OLC_**	5	48.65 ± 7.99	0.08 ± 0.01	47.7 ± 4.4	13.71 ± 3.08	3.06 ± 0.28	73.84 ± 5.57	3.59 ± 1.82	3.97 ± 2.44	6.87 ± 2.37
**mon SP-B**	4	131.10 ± 59.69	0.33 ± 0.16	62.7 ± 5.3	4.78 ± 2.01**	1.23 ± 0.45**	81.87 ± 1.38*	7.27 ± 2.25*	0.00 ± 0.00**	3.87 ± 1.94
**nat SF**	4	176.03 ± 68.91**	0.37 ± 0.21*	63.6 ± 6.2*	6.19 ± 1.73**	1.06 ± 0.62***	81.19 ± 1.43*	8.44 ± 1.10**	0.00 ± 0.00**	3.52 ± 0.79

**group**	**n (piglets)**	**PE [%]**	**SPH [%]**	**PUFA [%]**	**Usat FA [%]**	**TL 16:0 [%]**	**Tlfa 20:4 (AA) [%]**		**Tlfa 20:5 (EPA) [%]**	

**PPV_OLC_**	5	7.94 ± 3.63	3.80 ± 1.42	19.94 ± 1.39	40.34 ± 1.41	44.78 ± 1.73	5.54 ± 0.55		0.16 ± 0.03	
**monSP-B**	4	4.99 ± 1.21	2.02 ± 0.85	12.16 ± 3.76**	33.19 ± 3.27**	52.97 ± 3.78**	3.03 ± 0.71**		0.15 ± 0.02	
**nat SF**	4	0.47 ± 0.18	2.11 ± 0.81	11.52 ± 3.09**	35.76 ± 1.78*	50.24 ± 2.17**	2.58 ± 0.82***		0.29 ± 0.06**	

PPQ (phospholipid-to-protein ratio); Phospholipid classes are given as percent (w/w) of all phospholipids. LSA = large surfactant aggregates, given as percent (w/w) of all BALF phospholipids. PC (phosphatidylcholine); PG (phosphatidylglycerol); PS (phosphatidylserine); PI (phosphatidylinositol); PE (phosphatidylethanolamine); SPH (sphingomyelin); Fatty acids were dermined in the BAL total lipid fraction and are given as percent (w/w) of all fatty acids. PUFA (polyunsaturated fatty acids in the total lipid fraction); TL 16:0 (palmitic acid in the total lipid fraction). AA = Arachidonic acid, EPA = Eicospentaenoic acid

* p < 0.05 vs PPV_OLC_; ** p < 0.01 vs PPV_OLC_; ***p < 0.001 vs PPV_OLC_

§ p < 0.05 vs mon SP-B; §§ p < 0.01 vs mon SP-B

The relative content of large surfactant aggregates was increased in the surfactant-treated groups compared to PPV_OLC _(nat SF p = 0.06; mon SP-B p = 0.048). Both, total SP-B and SP-C concentrations were not statistically different between the experimental groups. Nevertheless, levels were significantly lower in the surfactant groups compared to the PPV_OLC _group (p < 0.001) when normalized to the total PL content of each sample.

### Interleukin and surfactant protein B and C mRNA expression analysis

Interleukin and SF mRNA expression was determined in the upper and lower left lung lobes. As there were no significant differences between the results obtained from the upper and lower left lung lobes, means were calculated and used for further analyses. Results normalized to β-Actin are represented in table 4. Normalization to the housekeeping gene HPRT confirmed the results.

**Table 4:** Pulmonary cytokine and surfactant protein mRNA expression (left lung lobe)

**group**	**n *(piglets)***	**IL-1β/A**	**IL-6/A**	**IL-8/A**	**IL-10/A**	**SP-B/A**	**SP-C/A**
**PPV_OLC_**	5	0.73 ± 0.51	1.27 ± 1.21	0.10 ± 1.3	0.34 ± 0.16	2.84 ± 2.17	0.82 ± 0.65
**mon SP-B**	4	0.35 ± 0.38	0.59 ± 0.78	0.03 ± 0.04	0.62 ± 0.32	4.05 ± 2.31	1.67 ± 0.87
**nat SF**	4	2.10 ± 1.77	3.96 ± 3.82	0.23 ± 0.22	0.67 ± 0.2	2.95 ± 1.27	1.09 ± 0.35

Normalization to β-Actin (A). Values are given as fold change (2^-ΔΔCt^). Means were calculated from values of upper and lower lung lobe.

Analyses of the pulmonary interleukin mRNA expression in the left lung revealed no significant differences between the study groups. Nevertheless, there was a tendency towards increased mRNA expression levels of the pro-inflammatory cytokines IL-1β, IL-6 and IL-8 in the nat SF group compared to mon SP-B and the PPV_OLC _group (p = 0.1; table 4). Furthermore, mRNA expression of the anti-inflammatory cytokine IL-10 showed a tendency towards increased levels in both surfactant groups compared to the PPV_OLC _group (p = 0.1; table 4). Furthermore, SP-B and -C mRNA expression were found to be not significantly different. However, SP-C mRNA expression was shown to be tendentially increased in the mon SP-B and nat SF groups compared to the PPV_OLC _group (table 4).

## Discussion

Respiratory failure often accompanies critical illness and determines morbidity in term neonates. Thus, evaluation of therapeutic strategies in neonatal ARDS-like lung disorders remains an important issue as demonstrated by a multitude of experimental and clinical studies [19,31]. Several concepts of treatment have been established in the last years. Under these, surfactant treatment is an established therapeutic option, as secondary surfactant deficiency may be causally related to the clinical picture and a potential cause for the impairment in lung function. Nevertheless, surfactant treatment is a very cost-intensive therapy and surely has its limitations due to adverse effects or the need for repetitive doses. Thus, conventional ventilation is often used as first-line management of the disease. If conventional ventilation strategies fail, further treatment regimes are searched for in order to minimize ventilator-induced lung injury [32]. Thus, ventilation following the open lung concept is often used as a rescue therapy. Nevertheless, there is still no consensus on ventilation strategy in these infants in combination or without surfactant administration until now [10]. Furthermore, different treatment regimes as exogenous surfactant administration under conventional ventilation or application of open lung ventilation strategies without surfactant replacement may result in differing effects on gas exchange, lung function, surfactant homoeostasis or pulmonary inflammatory processes and thus short and long term pulmonary outcome following neonatal ARDS.

In the present study, both treatment strategies investigated, administration of exogenous surfactant under conventional ventilation as well as application of the OLC, were found to be efficient in improving gas exchange and reconstituting lung function in neonatal ARDS. The effects on lung function and gas exchange have been confirmed for each treatment strategy in previous experimental studies [4,7,18].

To experimentally assess the effects of exogenous surfactant administration in neonatal ARDS compared to the OLC, a standard treatment regime using natural bovine surfactant has been chosen. However, surfactant preparations with varying protein and phospholipid contents have gained increasing interest in treatment of ARDS-like lung failure [11-13]. As native SP-B in humans is secreted in the alveoli predominantly in its dimeric form, modification of the dimeric structure leads to differences in the function of SP-B and is currently under further investigation [33,34]. Recently, modification of a natural surfactant preparation (SF-RI1) into a monomeric SP-B content has been demonstrated to improve biological activity compared to standard preparations in vivo [20]. Thus, the monomeric SP-B surfactant has been chosen as an alternative surfactant treatment in the present model.

Regarding the effects of the different treatment regimes applied on lung function and gas exchange in detail, the decrease of Pa_O2 _levels in the natural surfactant group at the end of the study period might reflect the need for repetitive doses of surfactant to achieve sustained treatment effects and may be a consequence of surfactant inactivation in the alveolar compartment [35]. Furthermore, levels of dynamic compliance did not reach initial values during the observation period and treatment effects declined to the end of the study period in the surfactant treated groups. In line with our findings, van Kaam and colleagues have shown a dose-dependency of the surfactant effect whereas a time-dependency with declining effects over time could be shown in the present study [36]. Besides the the need for repetitive or increased doses of exogenous surfactant the indicated effects may also be due to ventilator-induced lung injury under the conventional ventilation regime in the surfactant treated animals. Improvement of VEI over the study period was found to be most pronounced in the PPV_OLC _group, which may reflect the potential of this ventilation strategy to more equal recruitment of different parts of the lung. In contrast, surfactant is known to preferably reach lower lung lobes and its administration therefore often leads to inhomogenous recruitment and holds the risk of partial overdistension. However, ΔP values could be reduced in all study groups possibly leading to a reduction of shear stress and therefore traumatic lesions in the lung in the experimental ventilatory setting.

In order to define the impact of the treatment strategies applied on lavage-induced lung injury, surfactant homoeostasis and inflammatory processes, histologic patterns, cytokine and surfactant mRNA expression as well as phosholipid profiles of BALF were investigated.

Regarding histologic patterns, the evaluated score comprising different variables indicating the degree of lung injury showed no significant differences between the groups. Nevertheless, analyses showed alterations to pulmonary tissue, reflecting the delay of structural recovery compared to reconstitution of lung function and gas exchange after indcution of neontatal ARDS-like lung injury. The monomeric SP-B preparation revealed a tendency to improvement in lung structure, which may suggest the altered surfactant preparation to be effective in influencing surfactant homeostasis and thus the recovery in physiologic lung function.

Alterations to the surfactant system have been shown to closely reflect lung injury processes, especially alveolar type-II cell integrity and metabolism [25,28,37]. Furthermore, reconstitution of the surfactant system has been discussed as an important measure assessing different treatment regimes in ARDS-like lung disorders in children and infants. As expected, the phospholipid profile in BAL specimen showed a normalization of the phospholipid and fatty acid profile in both surfactant groups with an increase in concentrations of phosphatidylcholine and phosphatidylglycerol as well as the palmitic acid concentration in the total lipid fraction. In contrast, the phospholipid analyses of the PPV_OLC _group showed the typical profile for ARDS-like lung failure with a reduced recovery of phosphatidylcholine and phosphatidylglycerol, an increased relative content of polyunsaturated fatty acids, i.e. arachidonic acid and a decreased relative content of palmitic acid [28,37]. Furthermore, the relative content of large surfactant aggregates and the phospholipid-to-protein ratio in the BALF were significantly reduced in the PPV_OLC _group. These findings reflect a significantly reduced recovery of the surfactant system in the PPV_OLC _ventilated animals compared to the surfactant treated groups, potentially leading to short and long term consequences regarding lung injury processes and pulmonary function. Regarding the surfactant protein concentrations in the BALF, levels were comparabel in all study groups. In the case of SP-C, this may suggest a stabilization of type-II cell integrity and surfactant homeostasis under the indicated treatment regimes as previous studies showed significantly reduced SP-C levels in BAL specimen from conventionally ventilated ARDS patients [25]. An early recovery of alveolar type-II cell integrity and metabolism after lavage-induced lung injury may be further indicated by tendentially increased SPC mRNA expression in the in the surfactant treated groups.

Nevertheless, the combination of both treatment strategies will gain increasing interest as shown by latest studies [2,38]. Here, open lung ventilations strategies after exogenous surfactant administrations allowed reduction of both the opening and the closing pressures after some hours, which may be explained by a higher alveolar stabilization after recruitment maneuvers in combination with the effect of surfactant administration. Futhermore, the present study showed the relative SP-B and SP-C concentration after normalization to the total PL content of each sample, to be lower in the surfactant-treated groups. This may be assigned to a higher rate of ventilator-induced lung injury in the conventionally ventilated, surfactant treated animals, where higher ΔP values and tidal volumes compared to the PPV_OLC _group may led to an increased rate of shear stress to the lung. On the other hand, a shift in the phospholipid-to-protein ratio ratio may result from surfactant administration, as the indicated preparations contain relevant amounts of phopsholipids. Furthermore, limits of surfactant treatment in combination with a conventional ventilation strategy are demonstrated by cases with an absent response to exogenous surfactant administration as one animal in the natural surfactant and two animals in the monomeric SP-B group did not recover from lung failure and required an intensified ventilation regime.

In terms of pulmonary inflammatory processes, previous studies have been shown for the OLC ventilation to reduce signs of inflammation in BALF when compared to conventional ventilation strategies [4]. In the present study analyses of pulmonary pro-inflammatory cytokine mRNA expression showed no significant differences between the OLC and the surfactant treated groups. However, mRNA expression of the anti-inflammatory cytokine IL-10 showed a tendency towards increased levels in the surfactant groups compared to the PPV_OLC _group, possibly indicating anti-inflammatory or lung protective effects of exogenous surfactant. The tendency towards increased IL-1β, IL-6 and IL-8 mRNA expression in the natural surfactant group has also previously been shown in vivo for natural surfactant treated animals in a model of experimental meconium aspiration syndrome [9] and for endothelial cell activation in vitro [39] and may be explained by a higher concentration of arachidonic acid in the natural surfactant preparation.

In conclusion, we could show in a descriptive manner both treatment strategies, the administration of exogenous surfactant under conventional ventilation as well as the application of an intensified mechanical ventilation concept following the OLC to be efficient in improving lung function and gas exchange in neonatal ARDS. Nevertheless, administration of surfactant led to a more pronounced effect on gas exchange and compliance in the first hours, although improvement in oxygenation declined after 3 hours in the natural surfactant group. In contrast, improvement in VEI was found to be more evident in the PPV_OLC _group. Thus, differences between the surfactant and the PPV_OLC _groups regarding maintenance of the effects on lung function as well as surfactant homoeostasis and the pulmonary inflammatory balance may lead to pulmonary long-term consequences which should be addressed in further studies. Limitations of the study, that need to be addressed are the potential induction of lung injury processes by the initial phase of conventional ventilation, the induction of lung injury using repeated lavage procedures with each lavage volume potentially beyond total lung capacity leading to non-physiologic stretch as well as the application of relatively high tidal volumes in the PPVOLC group compared to previously published study settings applying OLC ventilation regimes. Changing ventilation strategies and modifying existing concepts reflect everyday clinical practice, but further studies are needed to address the impact of these variables on pulmonary outcome. As well clinical studies are needed in order to verify findings from animal studies. As the results indicate differing surfactant preparations to reveal distinct effects on lung function variables, further studies are needed to define an optimized surfactant composition.

## Competing interests

The authors declare that they have no competing interests.

## Authors' contributions

AH had responsibility for protocol development, performance of the experimental procedure as well as molecular analyses of the samples, preliminary data analysis and writing the manuscript. EA and TS participated in the performance of the experimental procedure. TB performed the real time PCR analyses. ME performed the histologic assessment. IR had primary responsibility for protocol development, performance of the experimental procedure and contributed to the writing of the manuscript. CR and AG performed the surfactant protein and phospholipid analyses and contributed to the writing of the manuscript. LG and JK supervised the design and execution of the study and contributed to the writing of the manuscript. All authors read and approved the final manuscript.

## Pre-publication history

The pre-publication history for this paper can be accessed here:


